# Phonological Units in Spoken Word Production: Insights from Cantonese

**DOI:** 10.1371/journal.pone.0048776

**Published:** 2012-11-07

**Authors:** Andus Wing-Kuen Wong, Jian Huang, Hsuan-Chih Chen

**Affiliations:** 1 The University of Hong Kong, Hong Kong S.A.R., China; 2 The Chinese University of Hong Kong, Hong Kong S.A.R., China; University of Leicester, United Kingdom

## Abstract

Evidence from previous psycholinguistic research suggests that phonological units such as phonemes have a privileged role during phonological planning in Dutch and English (aka the segment-retrieval hypothesis). However, the syllable-retrieval hypothesis previously proposed for Mandarin assumes that only the entire syllable unit (without the tone) can be prepared in advance in speech planning. Using Cantonese Chinese as a test case, the present study was conducted to investigate whether the syllable-retrieval hypothesis can be applied to other Chinese spoken languages. In four implicit priming (form-preparation) experiments, participants were asked to learn various sets of prompt-response di-syllabic word pairs and to utter the corresponding response word upon seeing each prompt. The response words in a block were either phonologically related (homogeneous) or unrelated (heterogeneous). Participants' naming responses were significantly faster in the homogeneous than in the heterogeneous conditions when the response words shared the same word-initial syllable (without the tone) (Exps.1 and 4) or body (Exps.3 and 4), but not when they shared merely the same word-initial phoneme (Exp.2). Furthermore, the priming effect observed in the syllable-related condition was significantly larger than that in the body-related condition (Exp. 4). Although the observed syllable priming effects and the null effect of word-initial phoneme are consistent with the syllable-retrieval hypothesis, the body-related (sub-syllabic) priming effects obtained in this Cantonese study are not. These results suggest that the syllable-retrieval hypothesis is not generalizable to all Chinese spoken languages and that both syllable and sub-syllabic constituents are legitimate planning units in Cantonese speech production.

## Introduction

It is widely accepted in the field of psycholinguistics that, during spoken word production, an abstract phonological plan is generated to guide subsequent articulatory processes. The processes of phonological plan formation have been referred to as phonological encoding [1]–[3]. However, this is where the consensus ends. The nature of phonological units and how they are processed during phonological encoding are still debatable.

To investigate the above-mentioned issues, many researchers have adopted the implicit priming (or form-preparation) paradigm introduced by Meyer [4]. In this paradigm, participants are asked to learn several sets of prompt-response word pairs and utter the response word upon seeing a prompt word. Response words within a block are either phonologically related (i.e., homogeneous) or unrelated (i.e., heterogeneous). Typically, participants' naming latencies are faster in homogeneous than in heterogeneous blocks if the response words share the same word-initial phoneme(s) together with the same stress pattern, but null effects are found if they share merely the same word-initial feature, word-final phonemes, or stress pattern [4]–[8]. These findings suggest that the phonological units activated at the beginning of phonological encoding are phoneme-based (aka the segment-retrieval hypothesis), and that the activated phonemes are associated with the word's metrical structure incrementally from left to right, consistent with the WEAVER (Word-form Encoding by Activation and VERification) model proposed by Levelt and colleagues [2], [9], [10].

Nevertheless, the findings referenced above are all from studies on Indo-European languages in particular Dutch and English. Using a similar implicit priming task, there is evidence from studies on distinct non-European languages, such as Mandarin Chinese [11], [12] and Japanese [13], showing that the functional units at the early stage of phonological encoding are language-dependent. Notably, J.-Y. Chen and colleagues demonstrated in a series of implicit priming studies that Mandarin speakers could benefit from similar word-initial syllables, regardless of supra-segmental features such as the lexical tone, in naming di-syllabic words [11], [14], [15]. However, null effects were repeatedly found when the response words (either di- or mono-syllabic) shared merely the same word-initial phoneme [11], [12]. Together with the convergent findings from other priming studies [16], [17] and speech error data [18], [19], these researchers have shown with compelling evidence that the segmental syllable (i.e., syllable as a chunk without the tone) has its own representation in Mandarin. To account for the implicit priming effects observed, they proposed that segmental syllables are the functional units (i.e., proximate units, according to O'Seaghdha, et al. [12]) addressed at the first place during phonological encoding so that advance preparation (e.g., syllable retrieval) is possible by fore-knowing the initial syllable but not merely the initial phoneme of the word in Mandarin [11]. And such proposal has been referred to as the syllable-retrieval hypothesis.

To account for these cross-language differences between Mandarin Chinese and Dutch or English, some possible accounts have been proposed [11]. For instance, Dutch and English adopt an alphabetic writing system in which the basic orthographic unit (i.e., a letter) corresponds roughly to a phoneme, whereas Chinese is logographic in which the fundamental orthographic unit (i.e., a character) maps directly onto a syllable. The differential orthographic experiences might therefore result in different phonological emphases [20], [21]. Furthermore, the size of syllable inventory is much larger in Dutch or English (>12000) than in Mandarin (∼400 segmental syllables), making it economically plausible to have syllable chunks stored as phonological units in Mandarin. In addition, dissimilar to many Indo-European languages in which syllable boundaries are sometimes ambiguous (e.g., /*m*/ in “format”) and re-syllabification is prevalent (e.g., /*k*/ is a coda in “talk” but an onset consonant in “talking”), the syllable boundaries in Mandarin are clear and fixed. Moreover, the phonology of Dutch or English is relatively more complex than that of Mandarin as there are more types of onsets and codas in the former than in the latter. Also, consonant clusters are prevalent in Dutch and English but are absent in Mandarin. The relatively simple syllabic structure in Mandarin might therefore favor the processing of syllable than phoneme, whereas the opposite is true for Dutch and English.

Nevertheless, studies on a different Chinese dialect, such as Cantonese, showed that sub-syllabic units also have a role to play during phonological encoding [22], [23]. Using a picture-word interference task, for instance, Wong and Chen reported that Cantonese speakers' picture naming responses were faster when the target (monosyllable with a Consonant-Vowel-Consonant structure) and distractor shared the same body (i.e., the CV component of a CVC syllable) or rhyme component than an unrelated control [23]. However, no significant effects were observed when the target and distractor shared merely the same initial phoneme [22]. Consequently, the authors suggested that in Cantonese speech preparation, sub-syllabic features have their unique representations and are important during phonological encoding but the effect of a single phoneme is not as salient as that in Indo-European languages such as Dutch and English [22], [23].

Although Wong and Chen observed significant priming of sub-syllabic components such as body and rhyme using a picture-word interference task, their findings do not refute the possibility that syllable units also have a unique role to play in Cantonese speech planning. In fact, Wong and Chen [22] observed that the priming effect of the syllable (without the tone) related condition was significantly larger than the rhyme-related condition. However, as the degree of segmental overlap across these two phonological conditions was not matched, it is unclear whether the stronger priming observed in the syllable-related condition was due to the unique contribution of syllable or to the relatively higher degree of segmental overlap between prime and target.

Since different speaker populations (Mandarin vs. Cantonese) and tasks (implicit priming vs. picture-word interference) were employed to investigate the processes of phonological encoding in spoken Chinese, it is unclear whether the presence [23] and absence [11] of sub-syllabic effects were due to task or language differences, or both. Similarly, it remains open whether the syllable-retrieval hypothesis previously proposed for Mandarin can be applied to other Chinese spoken languages. To address these issues, four experiments were conducted using the typical implicit priming paradigm with Cantonese-speaking participants. Following the previous work on Mandarin [11], the word pairs adopted in this Cantonese study were all di-syllabic words. The response words in a homogeneous block shared the same word-initial segmental syllable in Experiment 1, the same word-initial consonant in Experiment 2, and the same word-initial body in Experiment 3. If the syllable-retrieval hypothesis for Mandarin [11] is applicable to other Chinese dialects including Cantonese, one would expect to see significant priming effects only in a syllable-related condition (Exp.1) but not in a condition where response words shared similar sub-syllabic components (e.g., Experiments 2 and 3), similar to the case of Mandarin. Conversely, if phonological units smaller in size than a syllable are important during Cantonese phonological encoding, significant priming effects might also be observed in Experiment 2 or 3 of the present study. In addition, Experiment 4 was conducted to further investigate the possible roles of syllable and sub-syllabic components in Cantonese speech preparation using a similar implicit priming paradigm. In this experiment, the response words in a block shared either the same word-initial syllable (syllable-related condition) or the same word-initial body (body-related condition), or were unrelated (control condition). More importantly, the response words in the syllable-related condition all began with a CV syllable, whereas those in the body-related condition all began with a CVC syllable. By doing so, the degree of segmental overlap shared among the response words in the syllable-related and the body-related conditions was matched (i.e., the first consonant and vowel). If the syllable-retrieval hypothesis holds for Cantonese, one would expect to see significant priming in the syllable-related but not in the body-related condition. On the contrary, the segment-retrieval hypothesis predicts that comparable effects would be found across these two phonological conditions because the degree of segmental overlap possessed by the response words was the same. Alternatively, if both syllables and sub-syllabic components are legitimate planning units in Cantonese speech production, one would expect to see both syllable-related and body-related priming in Experiment 4 with the former being more robust than the latter.

## Results

Incorrect responses (< 6% of all original data) were excluded from subsequent response time analyses. Although the error rates were generally lower in the homogeneous condition (Mean = 4.7% in Exps. 1 to 3; Mean = 3.6% in Exp. 4) than in the heterogeneous condition (Mean = 5% in Exps. 1 to 3; Mean = 3.9% in Exp. 4), the difference was not significant in any of the four experiments (*p*s>.13). As the error rates were generally low and did not reveal any meaningful pattern, we will concentrate our discussions on the response time data.

Participants' response latencies in each of the first three experiments were submitted to a 2 (Context: Homogeneous vs. Heterogeneous condition) ×3 (Repetitions of the whole set of trials) within-subjects ANOVAs for both by participants (*F*_1_) and by items (*F*_2_) analyses. Dissimilarly, a 2 (Prime Type: Syllable-related vs. Body-related) ×2 (Context: Homogeneous vs. Heterogeneous condition) ANOVA was performed for the data from Experiment 4. Participants' mean naming latencies in various conditions of each experiment are shown in Table 1.

**Table 1:** Participants' mean naming latencies (M, in ms), standard errors (SE, in ms), and error rates (%) in various conditions of the four experiments.

				Context	
	Prime Type	Repetition		Homogeneous	Heterogeneous
Experiment 1	Syllable	1st	M	642	674
			SE	20	19
			Err (%)	5.4	6
					
		2nd	M	629	665
			SE	28	26
			Err (%)	6.4	5.4
					
		3rd	M	607	642
			SE	29	22
			Err (%)	4.5	5.6
					
					
Experiment 2	Onset	1st	M	725	724
			SE	22	17
			Err (%)	4.6	3.4
					
		2nd	M	704	713
			SE	21	19
			Err (%)	3.2	3.4
					
		3rd	M	695	690
			SE	22	17
			Err (%)	3.8	3.2
					
					
Experiment 3	Body	1st	M	703	709
			SE	27	28
			Err (%)	3.7	5.5
					
		2nd	M	682	694
			SE	30	27
			Err (%)	5.1	5.1
					
		3rd	M	675	697
			SE	26	26
			Err (%)	5.6	7.5
					
					
Experiment 4	Syllable	1st	M	611	644
			SE	12	13
			Err (%)	3.5	3.6
					
	Body	1st	M	637	653
			SE	13	11
			Err (%)	3.7	4.3

### Experiment 1: Word-initial segmental syllable

The main effect of Context was significant in both analyses, F1 (1, 11) = 8.01, p = 0.016; F2 (1, 15) = 15.52, p = 0.001, indicating that participants' responses were faster in the homogeneous condition than those in the heterogeneous condition. The effect of Repetition was significant in the item analysis only, F1 (2, 22) = 1.47, p = 0.25; F2 (2, 30) = 20.61, p<0.001. Furthermore, the Context x Repetition interaction was not significant, F1 (2, 22)<1; F2 (2, 30) = 1.13, p = 0.34, indicating that the effect of Context did not vary across trial repetitions.

### Experiment 2: Word-initial consonant

The main effect of Context was not significant, Fs<1. The effect of Repetition was significant in both analyses, F1 (2, 22) = 10.68, p<0.001; F2 (2, 30) = 28.18, p<0.001, indicating that participants' response times were decreasing over the trial repetitions. However, the Context x Repetition interaction was not significant, Fs<1.

### Experiment 3: Word-initial body

The main effect of Context was significant in both analyses, F1 (1, 11) = 6.61, p = 0.026; F2 (1, 15) = 8.14, p = 0.012, indicating that participants' responses were faster in the homogeneous condition than in the heterogeneous condition. The effect of Repetition was significant in the item analysis only, F1 (2, 22) = 2.24, p = 0.13; F2 (2, 30) = 6.48, p = 0.005. In addition, the Context x Repetition interaction was not significant, F1 (2, 22) = 1.33, p = 0.29; F2 (2, 30) = 2.46, p = 0.1.

### Experiment 4: Word-initial segmental syllable vs. Word initial body

The main effect of Prime Type was significant in the participant analysis only, F1 (1, 28) = 7.59, p = 0.01; F2 (1, 30) = 3.1, p = 0.089. The effect of Context was significant in both analyses, F1 (1, 28) = 16.7, p<0.001; F2 (1, 30) = 32.9, p<0.001, indicating that participants' responses were faster in the homogeneous condition than in the heterogeneous condition. Furthermore, the Prime Type x Context interaction was significant in both analyses, F1 (1, 28) = 4.43, p = 0.045; F2 (1, 30) = 4.28, p = 0.047. Subsequent paired-samples t-tests were conducted to examine the effect of Context in each Prime Type condition. Significant priming effects were found in both syllable-related (t1 [28]  = 4.67, p<0.001; t2 [15]  = 4.97, p<0.001) and body-related (t [28]  = 2.18, p = 0.038; t2 [15]  = 2.97, p = 0.01) conditions but the size of the former (33 ms) was apparently larger than the latter (16 ms).

## Discussion

### Summary of the results

Four experiments were conducted to investigate effective phonological units in Cantonese spoken word preparation using an implicit priming paradigm. The present results replicated those from previous Mandarin studies in which a significant facilitation in naming latency was observed when the response words in a block shared the same word-initial segmental syllable than when they did not (Exps.1 and 4), whereas no significant effects were found when the response words shared merely the same word-initial phoneme (Exp.2). Importantly, however, significant priming effects were obtained when the response words shared the same word-initial body in Experiments 3 and 4. To our knowledge, this is the first time a sub-syllabic effect was found in Chinese speech preparation using an implicit priming paradigm. In addition, a significant Prime Type x Context interaction was observed in Experiment 4, indicating that the priming effect observed in the syllable-related condition was larger than that in the body-related condition.

### Locus of the priming effects

The phonological effects obtained in a typical implicit priming task have been argued to be arising from the phonological level but not the subsequent phonetic encoding or motor execution stages [4], [6]. In the four present experiments, the response words within a block did not share the same lexical tone. Given that different articulatory programs are involved in producing the same segmental syllable with different tones, it is unlikely that the observed effects were due to the priming in motor execution. Furthermore, in Experiments 3 and 4 (body-related condition) of this study, although the response words in a homogeneous block shared the same word-initial body, they differed in the coda of their first syllable. In other words, the overlapping segments (i.e., the word-initial body) were situated at different phonetic contexts due to co-articulation. Therefore, it is unlikely that the priming effects were arising from the phonetic encoding stage because different phonetic plans were activated.

### Plausible explanations for the results

Experiments 1 and 2 of this Cantonese study replicated the major findings from previous Mandarin research [11], [12]: A significant syllable (without the tone) priming effect together with a null effect of the word-initial phoneme. These findings stand in marked contrast to those obtained from Dutch or English studies where a significant priming effect was also obtained with the word-initial phoneme [5], [7]. To account for these cross-language discrepancies, it has been proposed that phonemes are the functional units to be retrieved after lexical specification in languages such as Dutch and English (as assumed by the WEAVER model), whereas the corresponding units in Mandarin are segmental syllables [12]. Accordingly, Mandarin speakers can only be benefited from foreknowing the word's initial segmental syllable (i.e., syllable as a chunk) but not the word's initial phoneme in an implicit priming task.

The mentioned proposal for Mandarin can nicely account for the results of our first two experiments. However, it has difficulty to explain the results of our third experiment. Because the response words in this particular experiment did not share the same segmental syllable, no advance preparation should be possible according to the proposal for Mandarin. Furthermore, the body-related priming effect was replicated in our fourth experiment using a separate group of Cantonese-speaking participants and a slightly different set of materials, indicating that the effect is robust. However, according to J.-Y. Chen et al. [11], after the target segmental syllable has been retrieved in spoken word planning, its onset and rhyme components are spelled out and linearly associated with a metrical frame which specifies the tonal features of the word. Consequently, one might argue that in the case of Cantonese, the implicit priming task is also sensitive to such a segmental spell-out process, and the significant body-related priming was due to the advance linearization of the first two word-initial segments. If so, however, one needs to address why the locus of implicit priming is situated only at the syllable retrieval stage in Mandarin, but it can also be in the subsequent segmental spell-out process in Cantonese. Likewise, one needs to answer why an advance segment-to-frame association was possible in Experiments 3 and 4 even though a different tonal structure was required across trials.

Although the present results cannot be fully accounted for by the syllable-retrieval hypothesis, they are inconsistent with the segment-retrieval hypothesis which was previously proposed for Dutch and English as well. According to the segment-retrieval hypothesis, sub-syllabic units such as phonemic segments are the functional units to be retrieved after lexical specification [2]. Based on this view, the size of phonological priming observed in an implicit priming task is determined by the degree of segmental overlap among the response words [24]. Accordingly, one would expect to see comparable priming effects across the syllable-related and the body-related conditions in Experiment 4 because the degree of segmental overlap among the response words was kept constant across these two conditions (i.e., the two word-initial phonemes). However, the priming effect observed in the syllable-related condition was found much stronger than that in the body-related condition, as revealed by the significant Prime Type x Context interaction, indicating that the priming effects observed cannot be attributed to segmental priming alone. Instead, the relatively stronger syllable-related priming observed in Experiment 4 is consistent with the notion that the syllable unit has a unique role to play in phonological encoding as assumed by the syllable-retrieval hypothesis [11].

Note, however, that by using an odd-man-out paradigm, Cholin and colleagues [24] found evidence to suggest that the syllable units (i.e., syllabified phonological codes with metrical information specified) are the functional units at the *later* stage of phonological encoding in Dutch. Therefore, it is plausible that under certain conditions, significant syllable effects can also be observed even in languages which have been considered to rely mostly on segmental encoding. Nevertheless, the syllable effects observed in the current study in Cantonese (as well as those by J.-Y. Chen and colleagues in Mandarin) differ from that of Cholin et al. [24] in one important respect: The response words in the syllable-related condition in this study did not share the same lexical tone (i.e., metrical information), but those in Cholin et al. [24] shared the same metrical information. As such, the syllable (without the tone) priming effects observed in this study can only be attributed to an *early* stage of phonological encoding prior to the commencement of the prosodification processes.

The significant sub-syllabic priming (e.g., Exps. 3 and 4) as well as the distinct effects of segmental syllable (e.g., Exps. 1 and 4) observed in this Cantonese study suggest that both syllables (without the tone) and sub-syllabic units have their unique role to play at the early stage of Cantonese phonological encoding. To account for the present results, one might assume that the syllable (without the tone) units and their sub-syllabic constituents (e.g., phonemes) have distinct representations which are interconnected with each other, and are activated in parallel at the beginning of phonological encoding. The time needed for specifying the segmental content of the target depends on how fast the activation pattern between syllabic and sub-syllabic representations settles. Furthermore, this proposal assumes that the effect of a single phoneme (such as the onset consonant) is influenced by language differences. An individual phoneme might have a stronger influence in languages such as Dutch and English than in Cantonese or Mandarin. Consequently, the naming latencies of Dutch or English speakers could be facilitated by foreknowing the initial phoneme of the response words in an implicit priming task [4], [5], [25], but such benefit was not observed among speakers of Cantonese (Exp 2) or Mandarin [11], [12]. However, provided that there is sufficient degree of pre-activation at the sub-syllabic level (as in the case of the body-related condition), the time taken for settling the activation pattern between syllabic and sub-syllabic representations could be facilitated to a detectable extent (as reflected by the significant body-related priming effects in Exps. 3 and 4). In addition, since both the syllable unit and its sub-syllabic constituents could be pre-activated in a syllable-related condition (Exp. 1 and 4), the priming effect observed in such condition was found significantly larger than that in a body-related condition (Exp. 4), despite the fact that the amount of phonemic units being primed in these two conditions was the same.

Note that the above proposal is consistent with the findings from previous Cantonese studies using the picture-word interference paradigm [22], [23]. As mentioned in Introduction, Wong and Chen found that Cantonese speakers' picture naming responses were faster, relative to the control, when the target and distractor shared at least two similar phonemes, but not when they shared merely the same word-initial consonant. Furthermore, the priming effect observed in a syllable (without the tone) related condition was found significantly larger than that in a rhyme related condition. Taken these and the present findings together, they seem to suggest that both syllable and sub-syllabic components are legitimate processing units in Cantonese spoken word planning, but that the salience of a single phoneme is subject to the influence of language-specific properties.

Furthermore, existing theories of spoken word production assume that the phonological units to be retrieved following lexical specification, apart from the metrical features, are either segment-based [2] or syllable-based [11]. However, it remains largely open whether the two retrieval strategies can co-exist. In fact, to suggest that a general cognitive operation is sub-served by multiple sub-systems running in parallel is not uncommon in other areas of language processing. For instance, the dual route model of word reading proposed by Coltheart and colleagues [26] assumes that there are two ways to convert print to sound, namely a lexical route (i.e., mapping the word input with the corresponding entry in the lexicon) and a non-lexical route (i.e., decoding the pronunciation of the word by exploiting the grapheme-to-phoneme correspondences). Therefore, there is no clear reason why the segment-retrieval and the syllable-retrieval processes cannot exist together in phonological encoding. Indeed, as argued above, the present data can best be accounted for by assuming that these two retrieval strategies co-exist. Although the exact architecture of the phonological encoding processes might vary cross-linguistically due to language-specific properties, the present data suggest that both syllabic and sub-syllabic units are the functional units to be retrieved following lexical specification, at least in Cantonese.

As mentioned in Introduction, some plausible reasons have been proposed to account for the cross-language differences, in particular between Mandarin Chinese and Dutch or English, regarding the salience of an individual phoneme in speech planning. Similar to Mandarin, Cantonese is a tonal language adopting a logographic writing system. The size of syllable inventory is small (∼620 segmental syllables) relative to Dutch or English. Furthermore, the syllables in Cantonese are relatively simpler than those in Dutch or English as there is no consonant cluster in Cantonese, and the syllable boundaries are clear and fixed. In addition, the tones in Cantonese or Mandarin are lexically distinctive and the tonal features are realized mostly by the rhyme component of the syllables [27]. Consequently, speakers of Cantonese or Mandarin are more sensitive to the rhyme than to the onset consonant of a syllable because of the tonal information that the rhyme entails. All these proposed factors might contribute to the observed discrepancy in which a significant priming effect was found in Dutch or English speakers when the response words shared the same initial phoneme but not in Cantonese and Mandarin speakers using an implicit priming task [28].

Nevertheless, one issue related to the differences between Cantonese and Mandarin remains open. Specifically, a significant sub-syllabic priming effect was observed in the present Cantonese study (Exps. 3 and 4) but was not in the previous Mandarin studies. These results seem to suggest that these two speaker populations have slightly different emphases on the sub-syllabic level of processing in speech planning. Two plausible explanations are proposed for this cross-language difference. Firstly, although Cantonese is similar to Mandarin in many aspects, the phonology of Cantonese is relatively more complex than Mandarin (see [29] for details). For instances, Mandarin only has two types of coda (/n/ and /ng/), whereas Cantonese has six (/p/, /t/, /k/, /m/, /n/, and /ng/). The coda /ng/ can also appear as an onset in Cantonese, but not in Mandarin. Furthermore, Cantonese has six lexical tones (differentiated by both pitch height and contour), whereas Mandarin only has four (differentiated mostly by pitch contour) [30]. The differential demands on phonological analysis might promote speakers of different languages developing different degrees of sensitivity toward the fine-grained sub-syllabic units. Secondly, the participants in this Cantonese study were recruited from Hong Kong in which all of them started to acquire English as their second language as early as in the age of 4. The early exposure to an alphabetic language such as English might be a reason why Cantonese speakers were shown to be sensitive, albeit to a lesser extent than Dutch or English speakers, to the manipulation of sub-syllabic level in spoken word planning. However, these various proposed accounts are speculative which warrant further investigation.

### Significance of the present results

This study is the first showing that a sub-syllabic priming effect can be found in a Chinese language using the implicit priming task (Exps. 3 and 4), suggesting that sub-syllabic units have a role to play in Cantonese phonological retrieval, and that the syllable-retrieval hypothesis cannot be unrestrictedly generalized to other Chinese spoken languages. However, the present results also suggest that the syllable (without the tone) unit in Cantonese has its own representation and is important in phonological planning (Exps. 1 and 4). A plausible explanation for these findings assumes that there are unique representations for syllables and sub-syllabic components, and that these representations are activated in parallel at the beginning of phonological encoding. In addition, the present findings add to the evidence that the salience of an individual phoneme in speech preparation is language-dependent. Specifically, the effect of a single phoneme is much stronger in Dutch and English than in Cantonese and Mandarin. Some plausible reasons for the observed cross-language differences are discussed but warrant for future verification. Nevertheless, the present data show clearly that both syllabic and sub-syllabic units have a unique role to play in spoken word preparation. Conventional theories of speech production assume that either the segmental units or the entire syllables are processed following lexical specification, however, the present Cantonese data suggest a third possibility in which both syllabic and sub-syllabic units are activated at the beginning of phonological encoding.

## Materials and Methods

As the first three experiments employed the same experimental design and procedure but differed only in the specific stimuli used, a general method for Experiments 1 to 3 is described first, followed by a separate method section for Experiment 4.

### Experiments 1 to 3

#### Ethics statement

All experiments were approved by the Research Committee of the Department of Psychology at the Chinese University of Hong Kong. All participants gave informed consent before participating in the experiments.

#### Participants

Thirty-six healthy Cantonese-speaking undergraduates from the Chinese University of Hong Kong participated (12 in each experiment). They were paid (approx. US$ 7 each) for participation.

#### Apparatus

The stimuli (two-character Chinese words) were presented at the center of a 17-inch Flex Scan display monitor, with an IBM-compatible computer for controlling stimulus presentation. A prompt word shown in PMingLiu font was displayed at a size of approximately 1.4 cm×2.8 cm (approx. 2 degrees of visual angle) on each trial. Vocal responses were recorded via a microphone connected to the computer to the nearest millisecond by a voice onset relay.

#### Materials

Four sets of word pairs were included in each of the three experiments (see Table 2) with four word pairs in each set. In each pair, there were two semantically associated words (i.e., the prompt and the response word; e.g., 氧氣 “oxygen” and 呼吸 “breathing”). In the homogeneous condition, the initial syllable of the four response words in a block shared specific phonological contents (i.e., the segmental syllable, the onset consonant, and the body in Experiments 1, 2, and 3, respectively). Items in the heterogeneous condition were constructed by rearranging the word pairs from the homogeneous blocks such that the initial syllables of the four response words in a heterogeneous block were phonologically unrelated.

**Table 2:** Stimuli used in Experiments 1, 2, and 3.

Experiment 1: Syllable									
		Homogeneous							
	Set	/fu/		/gei/		/jan/		/si/	
Heterogeneous	1	氧氣	呼吸	際遇	機會	仇敵	恩怨	徒弟	師傅
		joeng5 hei3	**fu**1 kap1	zai3 jyu6	**gei**1 wui6	sau4 dik6	**jan**1 jyun3	tou4 dai2	**si**1 fu2
		*oxygen*	*breathing*	*turns in life*	*opportunities*	*enemy*	*dispute*	*apprentice*	*master*
	2	花開	富貴	數學	幾何	痛苦	忍耐	興趣	嗜好
		faa1 hoi1	**fu**3 gwai3	so3 hok6	**gei**2 ho4	tung3 fu2	**jan**2 noi6	hing3 cui3	**si**3 hou3
		*flowered*	*wealth*	*math*	*geometry*	*suffering*	*endurance*	*interest*	*hobby*
	3	電梯	扶手	新聞	記者	記憶	印象	日期	時間
		din6 tai1	**fu**4 sau2	san1 man4	**gei**3 ze2	gei3 yik1	**jan**3 zoeng6	jat6 kei4	**si**4 gaan3
		*escalator*	*handrail*	*news*	*reporter*	*memory*	*impression*	*date*	*time*
	4	貪官	腐敗	工匠	技巧	胎兒	孕婦	股票	市場
		taam1 gun1	**fu**6 baai6	gung1 zoeng6	**gei**6 hau2	toi1 yi4	**jan**6 fu5	gu2 piu3	**si**6 coeng4
		*venal officials*	*corruption*	*craftsman*	*skills*	*fetus*	*mother-to-be*	*shares*	*market*

Experiment 2: Onset									
		Homogeneous							
	Set	/f/		/g/		/j/		/s/	
Heterogeneous	1	花開	富貴	工匠	技巧	春天	雨水	廣告	宣傳
		faa1 hoi1	**f**u3 gwai3	gung1 zoeng6	**g**ei6 hau2	ceon1 tin1	**j**yu5 seoi2	gwong2 gou3	**s**yun1 cyun4
		*flowered*	*wealth*	*craftsman*	*skills*	*spring*	*rain*	*advertisement*	*promotion*
	2	天空	飛機	小說	故事	痛苦	忍耐	日期	時間
		tin1 hung1	**f**ei1 gei1	siu2 syut3	**g**u3 si6	tung3 fu2	**j**an2 noi6	jat6 kei4	**s**i4 gaan3
		*sky*	*plane*	*novel*	*story*	*suffering*	*endurance*	*date*	*time*
	3	蠟燭	火柴	宴會	嘉賓	詞鋒	銳利	幾何	數學
		laap6 zuk1	**f**o2 caai4	jin3 wui6	**g**aa1 ban1	ci4 fung1	**j**eoi6 lei6	gei2 ho4	**s**ou3 hok6
		*candle*	*match*	*banquet*	*guest*	*wording*	*critical*	*geometry*	*math*
	4	死亡	墳墓	治安	警隊	打扮	衣著	人民	社會
		sei2 mong4	**f**an4 mou6	zi6 on1	**g**ing2 deoi2	daa2 baan6	**j**i1 zeok3	jan4 man4	**s**e5 wui2
		*death*	*grave*	*public order*	*police*	*make up*	*dressing*	*people*	*society*

Experiment 3: Body									
		Homogeneous							
	Set	/daa/		/si/		/fu/		/ja/	
Heterogeneous	1	勇氣	膽量	功績	成就	尺碼	闊度	大門	入口
		jung5 hei3	**daa**m2 loeng6	gung1 zik1	**si**ng4 zau6	cek3 maa5	**fu**t3 dou6	daai6 mun4	**ja**p6 hau2
		*courage*	*bravery*	*merit*	*achievement*	*size*	*width*	*door*	*entrance*
	2	公園	單車	人像	攝影	金錢	奉獻	酒水	飲品
		gung1 jyun2	**daa**n1 ce1	jan4 zoeng6	**si**p3 jing2	gam1 cin4	**fu**ng6 hin3	zau2 seoi2	**ja**m2 ban2
		*park*	*bike*	*portrait*	*photography*	*money*	*offering*	*alcohol*	*beverage*
	3	考題	答案	餐廳	食物	衣著	款式	所有	一切
		hau2 tai4	**daa**p3 on3	caan1 teng1	**si**k6 mat6	ji1 zoek3	**fu**n2 sik1	so2 jau5	**ja**t1 cai3
		*question*	*answer*	*restaurant*	*food*	*dressing*	*style*	*possession*	*all*
	4	目標	達成	暴雨	閃電	長壽	福氣	汽車	引擎
		muk6 bui1	**daa**t6 sing4	bou6 jyu5	**si**m2 din6	coeng4 sau6	**fu**k1 hei3	hei3 ce1	**ja**n5 king4
		*target*	*accomplished*	*rainstorm*	*lightning*	*longevity*	*good fortune*	*vehicle*	*engine*

*Note.* The stimuli are presented in traditional Chinese. The English translations of the Chinese words are shown in italics. The number besides each syllable marking denotes the lexical tone of that syllable.

#### Design and procedure

The present design and procedure followed closely the ones used by Meyer [4] and J.-Y. Chen et al. [11]. In each experiment, each participant repeated the whole set of trials three times. In each repetition, there were both homogeneous and heterogeneous conditions, and the order of presentation was counterbalanced across participants. In each condition, there were four trial blocks with 16 trials in each. Each block consisted of four repetitions of the four word pairs in a set. The stimuli in a block (either homogeneous or heterogeneous) were randomly presented with the constraint that the same item did not repeat in consecutive trials. Altogether each participant received a sum of 384 trials (3 Repetitions ×2 Conditions ×4 Trial Blocks ×16 Trials).

A practice block was given prior to the start of the experimental session. The procedure of the practice block was the same as that of a trial block except that different stimuli were used. First, there was a self-paced study phase. Four pairs of semantically related words were shown on a computer screen. The prompt words were displayed on the left and the response words on the right. Participants were asked to study the four word pairs and told that they need to utter the corresponding response word upon seeing a prompt in the following session. After that, the prompt words were presented individually and oral responses were required. Incorrect responses were corrected by the experimenter immediately and the above steps were then resumed again until the participant produced all four correct responses.

Subsequently, a run of 16 trials (4 repetitions of the 4 prompt words) were presented to the participant. In each trial, a centered fixation cross (+) was first presented for 200 ms, followed by a blank of 600 ms, a prompt was then presented for 150 ms. Participants were asked to utter the corresponding response word as soon and as accurately as possible. Their naming latencies were measured from the onset of the prompt. The next trial began 200 ms after a response was detected or after a lapse of 1400 ms. A short break was given after each trial block and the whole experiment lasted for about an hour.

### Experiment 4

#### Ethics statement

Ethical approval was obtained from the Human Research Ethics Committee for Non-Clinical Faculties of the University of Hong Kong. Informed consent was obtained from all participants before commencing the experiment.

#### Participants

Twenty-nine healthy Cantonese-speaking undergraduates from the University of Hong Kong participated. They were paid (approx. US$ 7 each) for participation.

#### Apparatus

The stimuli were presented in the same way as in the previous experiments, and a similar set of equipments with comparable standards was used for stimuli display and data recording.

#### Materials

Eight sets of prompt-response word pairs (part of which were from Exps. 1 and 3) were included in this experiment (see Table 3) with four word pairs in each set. In four out of the eight sets of word pairs, the response words shared the same word-initial segmental syllable (syllable-related condition), whereas in the remaining four sets of word pairs, the response words shared the same word-initial body only (body-related condition). The response words in the syllable-related condition all began with a syllable having a CV (Consonant-Vowel) structure, whereas the response words in the body-related condition all began with a syllable with a CVC structure. Consequently, the response words in a syllable-related set and those in a body-related set similarly shared two identical word-initial phonemes (the first consonant + vowel), and the crucial difference between the two was that the phonological overlap in the former constituted a syllable-sized unit while the latter did not. In this way, the degree of segmental overlap shared among the response words in the syllable-related condition was matched with those in the body-related condition. Similar to the previous experiments, two controlled (heterogeneous) conditions were constructed by re-arranging the items from the syllable-related and the body-related homogeneous conditions correspondingly. The initial syllables of the response words in a heterogeneous block were phonologically unrelated.

**Table 3:** Stimuli used in Experiment 4.

Experiment 4: Syllable vs. Body									
Syllable-related		Homogeneous							
	Set	**/fu/**		**/si/**		**/co/**		**/waa/**	
Heterogeneous	1	氧氣	呼吸	興趣	嗜好	詩經	楚辭	中國	華人
		joeng5 hei3	**fu**1 kap1	hing3 cui3	**si**3 hou3	si1 ging1	**co**2 ci4	zung1 gwok3	**waa**4 jan4
		*oxygen*	*respiration*	*interest*	*hobby*	*Book of songs*	*Chinese classic*	*China*	*Chinese*
	2	花開	富貴	日期	時間	正月	初一	熱門	話題
		faa1 hoi1	**fu**3 gwai3	jat6 kei4	**si**4 gaan3	zing1 jyut6	**co**1 jat1	jit6 mun2	**waa**6 tai4
		*flowered*	*wealth*	*date*	*time*	*first month*	*first day*	*popular*	*topic*
	3	電梯	扶手	股票	市場	失敗	挫折	諷刺	挖苦
		din6 tai1	**fu**4 sau2	gu2 piu3	**si**6 coeng4	sat1 baai6	**co**3 zit3	fung3 ci3	**waa**1 fu2
		*escalator*	*handrail*	*shares*	*market*	*failure*	*setback*	*to mock*	*to ridicule*
	4	貪官	腐敗	徒弟	師傅	農夫	鋤頭	美術	畫家
		taam1 gun1	**fu**6 baai6	tou4 dai2	**si**1 fu2	nung4 fu1	**co**4 tau2	mei5 seot6	**waa**2 gaa1
		*venal officials*	*corruption*	*apprentice*	*master*	*farmer*	*hoe*	*fine arts*	*painter*
Body-related		Homogeneous							
	Set	**/daa/**		**/si/**		**/fu/**		**/ja/**	
Heterogeneous	1	勇氣	膽量	功績	成就	電器	風扇	大門	入口
		jung5 hei3	**daa**m2 loeng6	gung1 zik1	**si**ng4 zau6	din6 hei3	**fu**ng1 sin3	daai6 mun4	**ja**p6 hau2
		*courage*	*bravery*	*merit*	*achievement*	*electric appliance*	*fan*	*door*	*entrance*
	2	公園	單車	人像	攝影	顧客	服務	酒水	飲品
		gung1 jyun2	**daa**n1 ce1	jan4 zoeng6	**si**p3 jing2	gu3 haak3	**fu**k6 mou6	zau2 seoi2	**ja**m2 ban2
		*park*	*bike*	*portrait*	*photography*	*customer*	*service*	*alcohol*	*beverage*
	3	問題	答案	餐廳	食物	時裝	款式	所有	一切
		man6 tai4	**daa**p3 on3	caan1 teng1	**si**k6 mat6	si4 zong1	**fu**n2 sik1	so2 jau5	**ja**t1 cai3
		*question*	*answer*	*restaurant*	*food*	*fashion*	*style*	*havings*	*all*
	4	目標	達成	暴雨	閃電	名人	闊綽	汽車	引擎
		muk6 bui1	**daa**t6 sing4	bou6 jyu5	**si**m2 din6	ming4 jan4	**fut**3 coek3	hei3 ce1	**ja**n5 king4
		*target*	*accomplished*	*rainstorm*	*lightning*	*celebrity*	*ostentatious*	*vehicle*	*engine*

*Note.* The stimuli are presented in traditional Chinese. The English translations of the Chinese words are shown in italics. The number besides each syllable marking denotes the lexical tone of that syllable.

#### Design and procedure

The design and procedure of Experiment 4 was largely similar to that of the first three experiments except for the following aspects. First, both syllable-related and body-related conditions were included and tested by the same group of participants in Experiment 4, instead of by different groups of participants as in Experiments 1 and 3. Second, there was no repetition of the whole item set such that the potential influence of item repetition could possibly be minimized. In addition, the presentation order of the two phonological conditions was randomized across participants. Altogether each participant received a sum of 256 trials (2 Prime types ×2 Context conditions ×4 Blocks of trial ×16 Trials per block).
